# Immune responses following the first dose of the Sputnik V (Gam-COVID-Vac)

**DOI:** 10.1038/s41598-022-05788-6

**Published:** 2022-02-02

**Authors:** Chandima Jeewandara, Suranga Fernando, Pradeep Darshana Pushpakumara, Shyrar Tanussiya Ramu, Achala Kamaladasa, Banuri Gunasekara, Inoka Sepali Aberathna, Heshan Kuruppu, Thushali Ranasinghe, Shashika Dayarathne, Osanda Dissanayake, Nayanathara Gamalath, Dinithi Ekanayake, Jewantha Jayamali, Ayesha Wijesinghe, Madushika Dissanayake, Gayasha Somathilake, Michael Harvie, Saubhagya Danasekara, Deshni Jayathilaka, Helanka Dinesh Kumara Wijayatilake, Nihal Weerasooriya, Chinthaka Kekulandara, Lisa Schimanski, Pramila Rijal, Tiong K. Tan, Tao Dong, Alain Townsend, Graham S. Ogg, Gathsaurie Neelika Malavige

**Affiliations:** 1 Allergy Immunology and Cell Biology Unit, Department of Immunology and Molecular Medicine, Faculty of Medical Sciences, University of Sri Jayawardanapura, Nugegoda, Sri Lanka; 2 Ministry of Health Sri Lanka, Colombo, Sri Lanka; 3 MRC Human Immunology Unit, MRC Weatherall Institute of Molecular Medicine, University of Oxford, Oxford, UK; 4 Centre for Translational Immunology, Chinese Academy of Medical Sciences Oxford Institute, University of Oxford, Oxford, UK

**Keywords:** Adaptive immunity, Antimicrobial responses, Infection, Infectious diseases, Lymphocytes, Vaccines, Immunology, Microbiology, Molecular medicine

## Abstract

As the first dose of Gam-COVID-Vac, is currently used as a single dose vaccine in some countries, we investigated the immunogenicity of this at 4 weeks (327 naïve individuals). 88.7% seroconverted, with significantly lower seroconversion rates in those over 60 years (p = 0.004) and significantly lower than previously seen with AZD1222 (p = 0.018). 82.6% developed ACE2 receptor blocking antibodies, although levels were significantly lower than following natural infection (p = 0.0009) and a single dose of AZD1222 (p < 0.0001). Similar titres of antibodies were observed to the receptor binding domain of WT, B.1.1.7 and B.1.617.2 compared to AZD1222, while the levels for B.1.351 were significantly higher (p = 0.006) for Gam-COVID-Vac. 30% developed ex vivo IFNγ ELISpot responses (significantly lower than AZD1222), and high frequency of CD107a expressing T cells along with memory B cell responses. Although single dose of Gam-COVID-Vac was highly immunogenic, administration of a second dose is likely to be beneficial.

## Introduction

The COVID-19 pandemic continues to cause devastation throughout the world, with the mortality rates being highest in countries in the African, Asian and Latin American regions with poor vaccine coverage^1^. However, many countries in Europe and the USA, which experienced massive outbreaks and high mortality rates in 2020, now have low case-fatality rates, mainly by using vaccines that were developed using novel technologies, such as the mRNA vaccines and the adenovirus vector vaccines^2^. Several adenoviral vector vaccines have been developed and have undergone phase 3 trials and are widely used in many countries, such as AZD1222 (Vaxzevria/Covishield), Ad26.COV2.S developed by Janssen, Gam-COVID-Vac (Sputnik V) and CanSino COVID-19 vaccine (CanSioBIO)^3–5^.

Gam-COVID-Vac (Spuntik V) is a two dose COVID-19 vaccine, which comprises two replicant-deficient recombinant adenovirus vectors^6^. The first dose of the vaccine contains a recombinant adenovirus type 26 (rAd26-S) and the second dose a recombinant adenovirus 5 (rAd5-S), both carrying the full-length spike protein^6^. The use of two types of adenovirus vectors given 21 days apart, as prime and boost, was to overcome any pre-existing immunity to adenoviruses within a given population, while enhancing the immunogenicity of the vaccine^7^. A high efficacy rate of 91.6% was observed in their phase 3 trials, which was higher than reported for other phase 3 trials that used adenoviral vector vaccines^5,8^. Although Gam-COVID-Vac has not yet received emergency use authorization by the WHO, it is reported to be authorized by 30 countries^9^ and is used in Sri Lanka. However, recently the first dose of Gam-COVID-Vac (rAd26-S) was marketed by the authorities as a single-dose COVID-19 vaccine, which was claimed to have an efficacy of 78.6% to 83.7% among elderly individuals in Russia^10^. However, there are no published data regarding the real-world immunogenicity of the first dose of Gam-COVID-Vac, nor any data on antibody responses to SARS-CoV-2 variants of concern (VOCs). Although the seroprevalence of adenovirus 26 is less than the seroprevalence of adenovirus 5, the seroprevalence rates of these viruses vary widely in different populations^11,12^. Therefore, based on the seroprevalence rates of adenovirus 26 in a given population, the immunogenicity of a single dose vaccine using a human adenovirus vector may change.

Currently, while Gam-COVID-Vac is used in Sri Lanka, many individuals are only given the first dose of Gam-COVID-Vac. As there are no data regarding the seroprevalence of adenovirus 26 virus in Sri Lanka, it would be important to evaluate the immunogenicity of the first dose (rAd26-S) in a real-world scenario. Therefore, we studied antibody responses to the SARS-CoV-2 virus, antibodies to the receptor binding domain (RBD) of the ancestral Wuhan variant and other VOCs, ex vivo T cell responses and their functionality, and memory B cell responses in a large cohort of Sri Lankan individuals who received the first dose of the Gam-COVID-Vac. In addition, in order to compare the immunogenicity of a single dose of the rAd26-S with another adenovirus vector vaccine, we compared the immunogenicity of this vaccine with previously published data of AZD1222 single dose responses at 4 weeks following vaccination in Sri Lankan individuals^13^.

## Results

### Seroconversion rates to the first dose of Gam-COVID-Vac

Of the 388 individuals at 4 weeks post vaccination, 61 (15.7%) were seropositive and therefore, they were excluded from the analysis of seroconversion rates. During this period of 4 weeks post vaccination, none of the baseline seronegative individuals reported a symptomatic infection. Of the 327 individuals who were seronegative at baseline, 203 (62.1%) were females. The mean age was 50.3 years (range 20 to 83 years). The demographic details and comorbidities of these individuals are shown in Supplementary Table 1.

**Figure 1:**
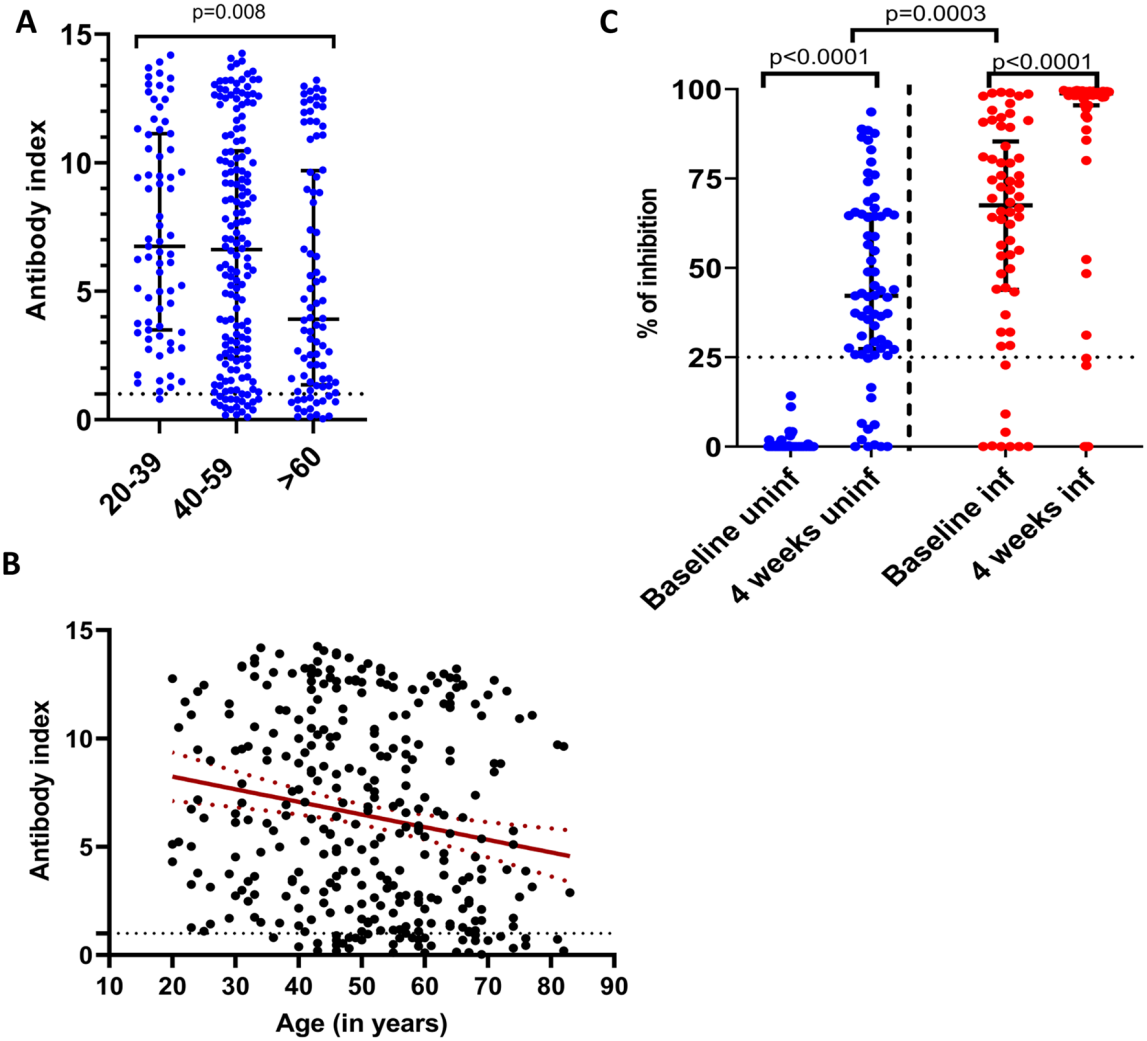
SARS-CoV-2 total antibodies and ACE2 receptor blocking antibodies in individuals 4 weeks after the first dose of Gam-COVID-Vac. The SARS-CoV-2 total antibody levels (antibody index values) were measured using the WANTAI SARS-CoV-2 Antibody ELISA assay in those between 20 to 39 years (n = 71), 40 to 59 years (n = 168) and 60 years (n = 88) (**A**) and the antibody levels were correlated with the age of individuals (n = 327), Spearman’s r = −0.21, p = 0.0001 (**B**). ACE2 receptor blocking antibodies were measured by the surrogate neutralizing antibody assay in a sub-cohort of baseline uninfected individuals (n = 69, blue) at 4 weeks and in a cohort of baseline infected individuals (n = 62, red), who were found to be seropositive for SARS-CoV-2 at the time of recruitment (**C**). The differences in the total antibody titres between different age groups was determined by the Kruskal–Wallis test. The differences in ACE2 receptor blocking antibodies at baseline uninfected and infected individuals were assessed by the Wilcoxon matched pairs signed ranked test. All tests were two sided. The error bars indicate the median and the interquartile ranges.

The overall seroconversion rates following a single dose of the vaccine was 88.7% (95% CI 85.2 to 92.1%). Seroconversion rates and the median antibody titres (given as the antibody index) are shown in Table 1. The seroconversion rates were significantly lower in older individuals (≥ 60 years of age) when compared to younger individuals (20–39 years of age) (Kruskal Wallis Chi Square = 11.137, p-value = 0.004). The antibody titres, as indicated by the antibody index, were significantly different in different age groups (p = 0.008, Fig. 1A) and significantly and inversely (Spearman’s r = −0.21, p = 0.0001) correlated with the age (Fig. 1B). The overall seroconversion rates for AZD1222 (93.2%)^14^ were significantly higher (p = 0.018) compared to Gam-COVID-Vac (88.7%). The seroconversion rates in the ≥ 60 age group (81.8%) were similar (p = 0.97) to levels seen following a single dose of AZD1222 at 4 weeks (81.6%)^14^.

**Table 1:** Seroconversion rates and antibody titres (given as the antibody index) 4 weeks following a single dose of the Gam-COVID-Vac.

Age categories	Positive N (%)	95% CI	Antibody index (median, IQR)
20–40, n = 71	70 (98.59%)	(95.85%, 100%)	6.7 (3.5 to 11.1)
40–59, n = 168	148 (88.10%)	(83.20%, 92.99%)	6.6 (2.4 to 10.5)
≥ 60, n = 88	72 (81.82%)	(73.76%, 89.88%)	3.9 (1.4 to 9.7)

75/327 (22.9%) had some comorbidity (e.g. diabetes, hypertension of chronic kidney disease). The seroconversion rates in those with comorbidities (84%, 95% CI 75.7 to 92.3%), was not different (p = 0.14) than in those did not have any comorbidities (90.1%, 95% CI 86.4 to 93.8%).

### ACE2 receptor blocking antibodies at 4 weeks following a single dose of Gam-COVID-Vac

ACE2 receptor blocking antibodies were determined using a surrogate neutralizing antibody assay (sVNT) as previously described^15^. A positive cut off value of 25% was considered as positive response for this assay in the Sri Lankan population, as previously described by us^16^. We measured ACE2 receptor blocking antibodies at paired samples at baseline uninfected individuals (n = 69) and baseline infected individuals (n = 62), at 4 weeks following vaccination. The individuals who were included in these analyses, were those who consented to give an additional volume of blood (12 ml vs 5 ml) as a larger volume was required for all the antibody and T cell assays.

57/69 (82.6%) individuals who were baseline uninfected gave a positive response with median values of 42.17% (IQR 27.3 to 64.9% of inhibition) (Fig. 1C). In comparison, 66/68 (97.1%) who received a single dose of AZD1222 gave a positive response to sVNT assay, with median antibody levels of 69.42 (IQR 54.09 to 81.54% of inhibition) as previously published^13^. We also compared the ACE2 receptor blocking antibody levels with baseline antibodies of those who were found to be infected, and in another cohort of 36 individuals who had varying severity of natural infection^17^. While the ACE2 receptor antibody levels were similar in the two naturally infected cohorts, the ACE2 receptor blocking antibodies were significantly lower in the naïve individuals who received 1 dose of the Gam-COVID-Vac compared to baseline seropositives (p = 0.0003), and those who were naturally infected (p = 0.0009) (Fig. 1B). In contrast, those who had a single dose of AZD1222 had similar levels of ACE2 receptor blocking antibodies as those following natural infection (p = 0.7). Those who had a single dose of AZD (median 69.4, IQR 54.1 to 81.5) had significantly higher levels (p < 0.0001) than those who received a single dose of Gam-COVID-Vac.

Of the baseline infected individuals, 9/62 (14.5%) did not have ACE2 receptor antibodies at baseline. However, the ACE2 receptor blocking antibodies significantly increased (p < 0.0001) from baseline (median 67.1, IQR 43.9 to 85.4% of inhibition) to 4 weeks following the vaccine (median 98.7, IQR to 95.5 to 99.3% of inhibition (Fig. 1B). However, 5/9 individuals who were found to be previously infected (gave a positive response by the Wantai total antibody assay), failed to develop ACE2 receptor blocking antibodies following vaccination.

### Antibodies to the RBD of the ancestral SARS-CoV-2 virus and variants of concern

We measured antibodies to the RBD of the ancestral variant (WT), B.1.1.7, B.1.351 and B.1.617.2 as previously described using the HAT assay^18^. A HAT titre of 1:20 was considered as the cut-off value for a positive response as previously described by us^19^. The assay was carried out on baseline uninfected individuals (n = 69) and baseline infected individuals (n = 62); these were the same individuals used to assess ACE2 receptor blocking antibodies. The levels were compared with our previously published data^13^ for those who received the AZD1222 at 4 weeks and additional assays were done for RBD antibodies for B.1.617.2 in this cohort as we had not assessed these antibodies previously.

**Figure 2:**
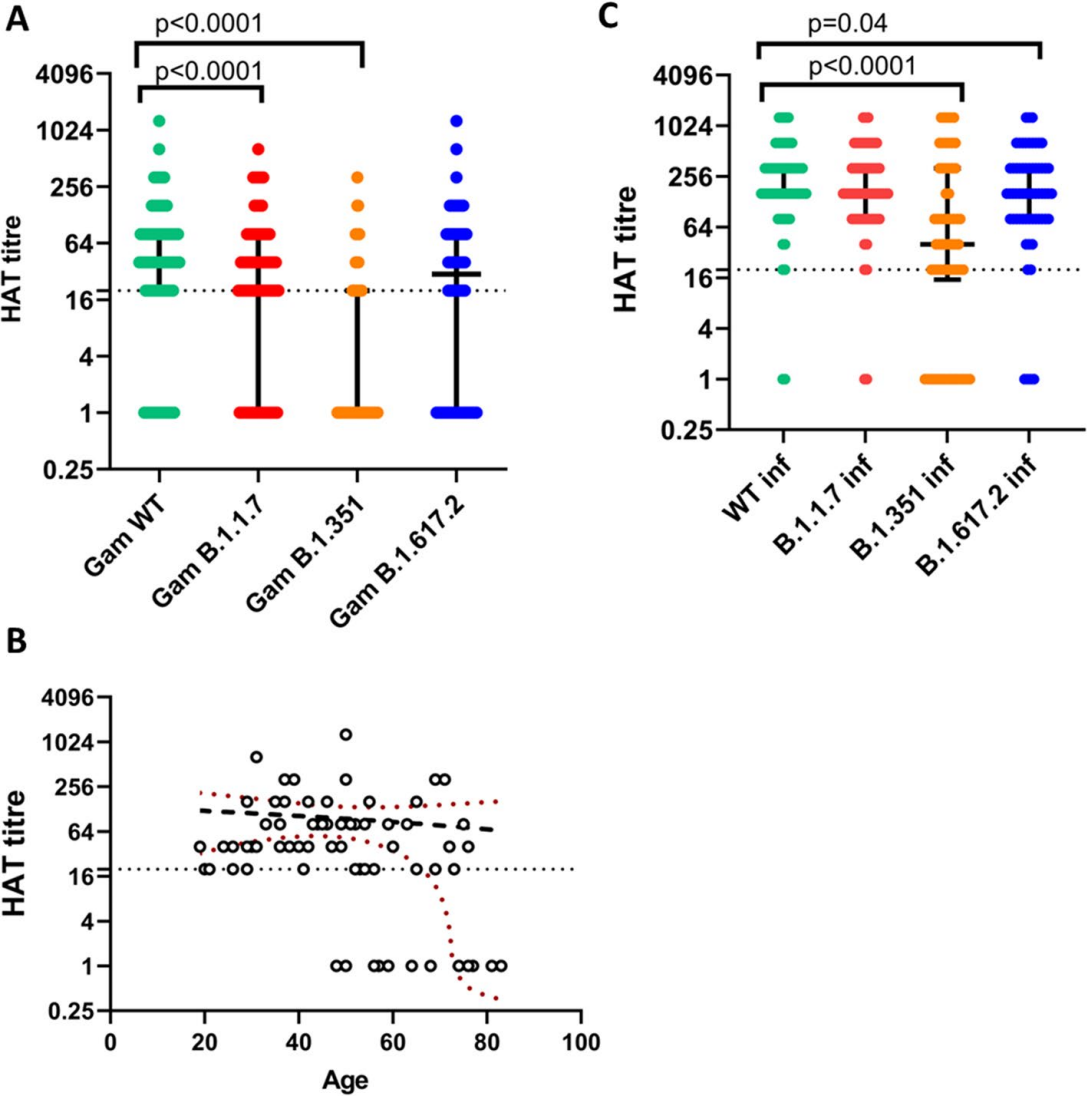
Antibodies to the RBD of SARS-CoV-2 Ancestral Wuhan (WT) virus and the variants of concern by the haemagglutination test (HAT). Antibodies to the RBD were measured by HAT in previously uninfected individuals at 4 weeks following a single dose of Gam-COVID-Vac (n = 69) for the WT, B.1.1.7, B.351.1 and B.1.617.2 (**A**) and in previously infected individuals (n = 62), at 4 weeks to WT, B.1.1.7, B.1.351 and B.1.617.2 (**B**). The age was correlated with the HAT titres for the WT (Spearman’s r = −0.26, p = 0.03). Wilcoxon matched pairs signed ranked test was used to find out the differences in HAT titres in baseline uninfected and infected individuals for WT and the VOCs. All tests were two sided. The error bars indicate the median and the interquartile ranges.

Of those who were baseline seronegative, 56/69 (81.2%) responded to RBD of WT, 49/69 (71.0%) to B.1.1.7, 25/69 (36.2%) to B.1.351 and 44/69 (63.8%) to B.1.617.2 (Fig. 2A). The RBD HAT titres were significantly lower for the WT (p < 0.0001), B.1.1.7 (p < 0.0001), B.1.351 (p = 0.001), B.1.617.2 (p = 0.0003) compared to titres of convalescent sera of naturally infected individuals (n = 36)^17^. The mean HAT titres for Gam-COVID-vac were similar to the WT (95.6 vs 94.1), B.1.1.7 (62.9 vs 48.8) and for B.1.617.2 (73.9 vs 70.4), compared to our previously published data following a single dose of AZD1222. However, the HAT titres to the RBD of B.1.351 (mean 23.2, SD ± 52.2) were significantly higher (p = 0.006), in comparison to AZD1222 (mean 5.6, SD ± 14.6). The geometric means of the HAT titres following the Gam-COVID-Vac for WT RBD was 5.4-fold lower, B.1.1.7 8.2 folds lower, 0.5 folds lower for B.1.351 and 3.9 folds lower for B.1.617.2 compared to levels seen following natural infection. The HAT titres for the RBD of the WT significantly and inversely declined with the age of the individual (Spearman’s r = −0.26, p = 0.03) (Fig. 2B).

HAT assays for the RBD of WT and VOCs were also carried out on those who were baseline infected. Except for 2 individuals, all others developed antibodies to the RBD of WT and B.1.1.7. Although the HAT titres to the WT and B.1.1.7 were similar to the levels seen in naturally infected individuals, the vaccinees (Gam-COVID-Vac) had significantly higher responses to B.1.351 (p < 0.0001) and B.1.617.2 (p = 0.03) (Fig. 2C).

### Ex vivo IFNγ ELISpot responses and intracellular cytokine responses to Gam-COVID-Vac

IFNγ ELISpot responses were done in 40 individuals (who were seronegative at baseline) at 4 weeks from receiving the first dose of Gam-COVID-Vac. Since the cut-off value of a positive response was considered to be mean ± 2SD, the threshold for a positive response was set at 220 spot forming units (SFUs)/1 million PBMCs. Accordingly, 12/40 (30%) had a positive response to S1 pool of peptides and 11/40 (27.5%) had a positive response to S2. The positive threshold for the AZD1222 was set at 302 SFU/1 million PBMCs. The proportion of individuals who responses to AZD1222 was higher for S1, with 42/68 (61.7%) of individuals S1 pool of peptides, while the responses to S1, 21/68 (30.8%) was similar.

**Figure 3:**
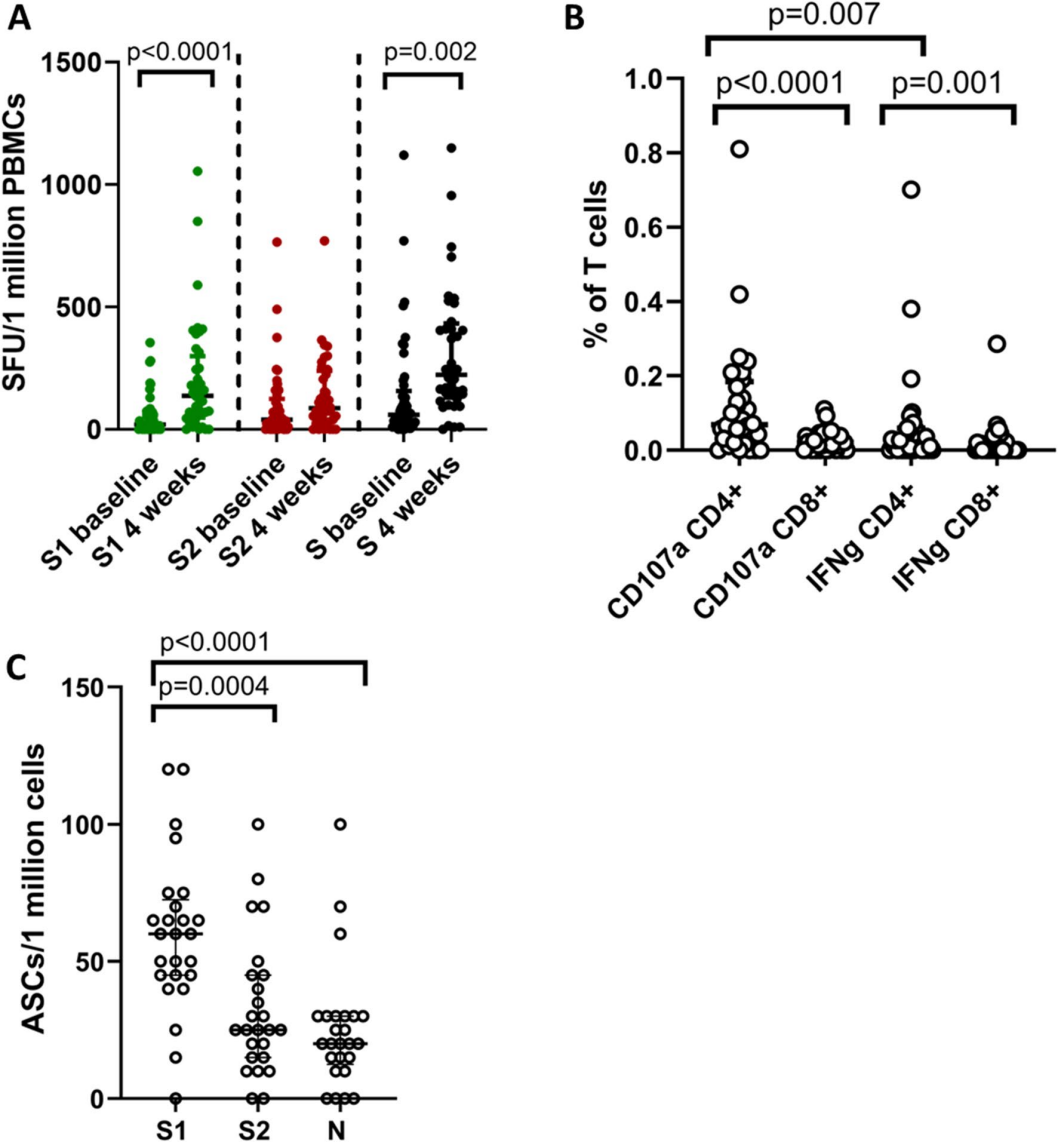
SARS-CoV-2 specific T cell and B cell responses in individuals who received the Gam-COVID-Vac. Ex vivo IFNγ ELISpot assays were carried out at 4 weeks (individuals who were vaccinated from Gam-COVID-Vac) (n = 40) in baseline uninfected individuals for overlapping peptides of spike protein, which were in two pools S1 and S2 (**A**). Intracellular cytokine staining was used to determine CD107a expression and IFNγ production at 4 weeks (n = 32) in CD4+ and CD8+ by flow cytometry (**B**). The number of antibody secreting B cells (ASCs) were determined at 4 weeks (n = 25) by doing B cell ELISpot assays (**C**). Wilcoxon matched pairs signed ranked test was used to find out the differences in ex vivo ELISpot responses for S1 and S2, and differences in CD107a expression and IFNγ production by CD4+ and CD8+ T cells and for B cell ELISpot assays. All tests were two sided. The error bars indicate the median and the interquartile ranges.

The responses to S1 pool of peptides significantly increased (p < 0.0001) from baseline to 4 weeks (median 137.5, IQR 48.7 to 298.8 SFU/1 million PBMCs), while a significant increase (p = 0.23) was not seen for the S2 pool of peptides (median 87.5, IQR 41.3 to 240 SFU/1 million PBMCs) (Fig. 3A). The ELISpot responses for neither S1 nor S2 correlated with the age of the individuals. Individuals had significantly higher (p < 0.0001) ex vivo IFNγ ELISpot responses to the S1 pool of peptides following the AZD1222^13^ compared to the first dose of Gam-COVID-Vac, and for the S2 pool of peptides (p = 0.01).

Intracellular cytokine assays (ICS) was carried out in a subset of the above individuals (n = 32) in whom IFNγ ELISpot responses were carried out due to limitations in the number of cells. A relative high frequency of CD107a expressing CD4+ (median 0.069, IQR 0.022 to 0.18% of CD4+ T cells) and CD8+ T cells (median 0.021, IQR 0 to 0.045% of CD8+ T cells) were seen with CD4+ T cells expressing significantly more CD107a (p < 0.0001) (Fig. 3B). IFNγ production was also predominantly by CD4+ T cells (median CD4+ T cells 0.01 vs 0 from CD8+ T cells), which was significant (p = 0.001). Spike protein specific CD4+ T cells expressed significantly more CD107a (p = 0.007) than IFNγ. No comparison could be carried out with AZD1222 as we had not carried out ICS for those who received the first dose of AZD1222 at the 4 weeks’ time point.

### Memory B cell responses to the Gam-COVID-Vac

Antibody secreting memory B cell responses were assessed by B cell ELISpot assays for S1, S2 and N recombinant protein at 4 weeks (n = 25) following vaccination in baseline seronegative individuals. A positive response was defined as mean ± 2 SD of the background responses. Accordingly, a cut-off of 22.1 antibody secreting cells (ASCs)/1 million cells was considered as the positive threshold for S1 protein, 25.2 for S2 and 10.3 for N protein. 23/25 individuals had a positive response to S1, 11/25 and 19/25 gave a positive response to S2 and N protein respectively, in those who were baseline seronegative. However, the responses for S1 (median 60, IQR 45 to 72.5 ASCs/1 million cells) were significantly higher than (p = 0.0004) for S2 (median 25, IQR 15 to 45 ASCs/1 million cells) and significantly higher than (p < 0.0001) for N (median 20, IQR 12.5 to 30 ASCs/1 million cells) (Fig. 3C).

## Discussion

We have investigated the immune responses of the first dose (rAd26-S) of the Gam-COVID-Vac, which is also known as Sputnik light, in a Sri Lankan population, 4 weeks after receiving the first dose and compared the immune responses with our previously published data following AZD1222, which is another adenovirus vector vaccine. The first dose of Gam-COVID-19 vaccine was shown to induce an overall seroconversion rate of 88.7%, with significantly lower seroconversion rates (81.8%) and SARS-CoV-2 specific antibody levels in individuals over 60 years of age. It induced ACE2-receptor blocking antibodies in 82.6% of individuals and the levels were significantly lower than levels seen following a single dose of AZD1222 and those following natural infection. Therefore, the overall antibody responses following a single dose of Gam-COVID-Vac appear to be significantly lower than those following a single dose of AZD1222, especially in older individuals in a Sri Lankan population^13^. Older individuals are known to have impaired innate and adaptive immune responses to new antigens due to multiple factors such as compromised antigen presentation, T cell exhaustion and a limited B cell receptor repertoire in memory B cells leading to development of antibodies with poor affinity and avidity^20^. Therefore, due to the reduced immunogenicity of Gam-COVID-Vac, and due to the rapid decline in immune responses to some COVID-19 vaccines^21^, different dosing schedules and booster dosing schedules may have to be implemented in older individuals.

In the phase 1/2 studies, it was shown that the vaccine only induced neutralizing antibodies (Nabs) in 61.1% of individuals at 28 days although the antibody titres were comparable to those following natural infection^6^. Although the ACE2 receptor blocking antibodies are not a direct measure of Nabs, they have shown to be a surrogate indicator of Nabs^15,22^. Nabs have been shown to be highly predictive of protection against SARS-CoV-2 infection^23^, and those with lower Nabs have shown to associate with breakthrough infection in vaccinated health care workers^24^. Although the AZD1222 reported an efficacy rate of 76% up to 90 days following a single dose of the vaccine for the ancestral SARS-CoV-2 virus^25^, the efficacy of a single dose reduced to 30% for B.1.617.2^26^. Therefore, given that the ACE2 receptor blocking antibody levels were significantly lower than following a single dose of AZD1222 and since the proportion of individuals who developed ACE2 receptor blocking antibodies were also significantly lower, especially in the older age groups. A single dose of Gam-COVID-Vac may possibly have a reduced efficacy than AZD1222 in Sri Lanka, and so may benefit from administration of the second dose.

At the end of 4 weeks following the single dose of Gam-COVID-Vac, 81.2% had antibodies to the RBD of the WT detected by HAT, similar to levels seen following a single dose of AZD1222^13^. However, the proportion of individuals who developed antibodies were less than those reported in the phase 1/2 study, in which 100% of the study participants developed antibodies to the RBD by 21 days^6^. These differences could be due to the differences in the assays used, ages of the individuals included (we had included individuals > 60 years of age), sample size (n = 18 in the published study compared to n = 69 in this study) and possible pre-existing antibodies to Ad26 reducing vaccine immunogenicity in Sri Lanka^27^. The lower RBD antibodies to the WT and ACE2 receptor blocking antibodies in our cohort of patients, especially in those who were > 60 years of age, in comparison to the published data, could be due to the differences in the seropositivity rates for adenovirus 26 in different age groups in different populations^12^. It was previously shown that individuals who were seropositive for adenovirus 5, had a higher risk of acquiring HIV following immunization with an HIV vaccine with adenovirus 5^28^. This increased risk of acquiring HIV was thought to be due to reduced HIV specific CD4+ T cell responses and a reduced breadth of CD8+ T cell responses, in vaccine recipients who were seropositive for adenovirus 5^29^. As pre-existing anti-vector immunity is likely to dampen the immune responses to vaccines, it would be important to study the seroprevalence of antibodies to the adenovirus vector used in such vaccines, in order to further understand the effect of a vaccine when rolling it out in a population.

The antibody responses to the RBD of the WT, B.1.1.7 and B.1.617.2 following the first dose of Gam-COVID-Vac were similar to that of AZD1222, but significantly lower than antibody levels seen following natural infection, although antibodies to the RBD of B.1.351 were significantly higher than AZD1222. As AZD1222 was shown to have a reduced efficacy for B.1.351^30^, given the higher magnitude and frequency of antibodies to the RBD of B.1.351, Gam-COVID-Vac may be more effective than AZD1222 in countries, which have outbreaks due to B.1.351, although further clinical studies are required. However, since many of the SARS-CoV-2 variants such as B.1.1.7 and B.1.351 are on the decline, the immune responses to the new emerging sub lineages of delta should be continuously monitored.

Although Nabs are thought to be correlated with prevention of breakthrough infections following vaccination and an important correlate of protection^23^, a robust T cell response is also associated with development of milder clinical disease^31^. Gam-COVID-Vac induced a high frequency of ex vivo IFNγ ELISpot responses to spike protein peptides in 30% of individuals, and a high frequency of CD107a producing CD4+ and CD8+ T cells along with memory B cell responses. Interestingly, both CD107a and IFNγ production was predominantly by the CD4+ T cell subset of T cells, which is similar to what was seen following a single dose of the AZD1222^32^. However, the frequency of ex vivo IFNγ ELISpot responses and the proportion of individuals who responded to the spike protein overlapping pool of peptides was significantly lower than for AZD1222^13^. The Gam-COVID-Vac induced ASCs, responding to S1, S2 and N recombinant proteins. Although 19/25 individuals responded to the N protein of SARS-CoV-2, these responses were of very low frequency and were several folds lower than the responses to S1 subunit. The frequency of N protein specific ASCs were also several fold lower than what we previously observed for the Sinopharm/BBIBP-CorV, which is an inactivated vaccine and therefore, induces immune responses to the N protein^17^. Therefore, these low frequency memory T cell responses are likely to be due to pre-existing cross reactive memory B cell responses to other seasonal coronaviruses and are unlikely to be SARS-CoV-2 specific^33^.

In summary, the Gam-COVID-19 first dose (rAd26-S) induced seroconversion rates in 88.7% of individuals 4 weeks following the vaccine, with significantly lower seroconversion rates in the elderly in a Sri Lankan population. ACE2 receptor blocking antibody responses were seen in 82.6% of individuals, with levels significantly lower than after a single dose of AZD1222 and following natural infection. While the antibody responses to the RBD by HAT were similar to AZD1222, the ex vivo IFNγ ELISpot responses were significantly lower. The findings suggest that the two Gam-COVID-19 doses using heterologous adenoviruses may be of particular importance in certain populations. This data further highlights the importance of studying the prevalence of different adenoviruses and how pre-existing immunity affects the immune responses to different adenovirus vector vaccines. This information is viral in order to determine timing and targeting additional doses of COVID-19 vaccines.

## Methods

### Study participants

388 adult individuals from Sri Lanka, were recruited at the time they received the first dose of the vaccine following informed written consent. Blood samples were obtained at baseline (at the time of recruitment) to determine SARS-CoV2 seropositivity, and at 4 weeks from receiving the first dose of the vaccine. Individuals who were between the ages of 20 to 39 years were considered as younger individuals, while those who were > 60 years of age, were considered as older individuals. The presence of comorbid illness such as diabetes, hypertension was recorded. The SARS-CoV-2 specific antibody assays were carried out in all the individuals, while certain assays were done in a sub cohort (see below). We also compared the ACE2 receptor blocking antibodies, antibodies to the RBD of different variants detected by HAT and ex vivo IFNγ ELISpot responses in individuals 4 weeks after a single dose of AZD1222 (Covishield) vaccine (n = 69)^13^, and with those who were naturally infected (n = 36) as previously reported by us^17^. Overall seroconversion rates for Gam-COVID-Vac first dose was also compared with AZ first dose at 4 weeks, using our previously published data^14^.

Ethics approval was obtained from the Ethics Review Committee of University of Sri Jayewardenepura (COVID 01/21). All research was performed in accordance with relevant guidelines/regulations, and informed written consent was obtained from all participants.

### SARS-CoV-2 specific total antibodies, ACE-2 receptor blocking antibodies and antibodies to the RBD of SARS-CoV2 variants

Antibodies to SARS-CoV-2 were detected by Wantai SARS-CoV-2 antibody ELISA (Beijing Wantai Biological Pharmacy Enterprise, China), which detects IgM, IgG and IgA antibodies. Surrogate virus neutralization test (sVNT)^15^ was used to detect ACE2 receptor blocking antibodies as previously described^16^. Inhibition percentage ≥ 25% in a sample was considered as positive for ACE2 blocking antibodies. This assay was found to be 100% specific for measuring ACE2 blocking antibodies in the Sri Lankan population^16^.

### Haemagglutination tests for detection of antibodies to the RBD in WT and SARS-CoV-2 variants

For the HATs, sera were doubling-diluted in 50 μl PBS in V bottomed 96 well plates, 50 μl of ~ 1% v/v O-ve red cells were added, followed by 50 μl of the relevant IH4-RBD reagent diluted to 2 μg/ml (100 ng/well). Plates were incubated for 1 h at RT, tilted for ~ 20 s to allow a red cell “teardrop” to form, photographed and read by eye. The RBD-specific antibody titre for the serum sample was defined by the last well in which the complete absence of “teardrop” formation was observed. The HAT titration was performed using 7 doubling dilutions of serum from 1:20 to 1:1280, to determine presence of RBD-specific antibodies. A titre of 1:20 was considered as a positive response, as previously determined by us^19^. The IH4-RBD reagents for each VOC were standardized by titration with the monoclonal antibody EY-6A^18,34^ that binds to a conserved epitope common to all variants. A 20 μg/ml solution of EY-6A titrated equally with a standard (2 μg/ml) solution of each of the new IH4-RBD reagents. All were therefore added as 50 μl from a 2 μg/ml stock solution (100 ng/well) as described^18^.

### Ex vivo ELISpot assays

Ex vivo IFNγ ELISpot assays were carried out using freshly isolated peripheral blood mononuclear cells (PBMC). Two pools of overlapping peptides named S1 (peptide 1 to 130) and S2 (peptide 131 to 253) covering the whole spike protein (253 overlapping peptides) were added at a final concentration of 10 µM and incubated overnight as previously described^35,36^. 100,000 cells/well were added, PHA was included as a positive control of cytokine stimulation and media alone was applied to the PBMCs as a negative control. All peptide sequences were derived from the wild-type consensus and were tested in duplicate.

Briefly, ELISpot plates (Millipore Corp., Bedford, USA) were coated with anti-human IFNγ antibody overnight (Mabtech, Sweden). The plates were incubated overnight at 37 °C and 5% CO_2_. The cells were removed, and the plates developed with a second biotinylated Ab to human IFNγ and washed a further six times. The plates were developed with streptavidin–alkaline phosphatase (Mabtech AB) and colorimetric substrate. The spots were enumerated using an automated ELISpot reader (AID Germany). Background (PBMCs plus media alone) was subtracted and data expressed as number of spot-forming units (SFU) per 10^6^ PBMCs. A positive response was defined as mean ± 2 SD of the background responses. An example of a ex vivo ELISpot assay for S1, S2, is shown in Supplementary Figure 1.

### Intracellular cytokine staining

Intracellular cytokine staining was carried out in freshly isolated PBMCs. PBMCs were incubated with CD107a FITC (Biolegend, USA) for 30 min in RPMI 1640 and 10% heat inactivated human serum (Sigma Andrich). Cells were stimulated with overlapping peptides pool of SARS-CoV-2 spike protein for 2 h at 1 mM concertation before adding monensin (Biolegend, USA). The PBMC were incubated for a further 14 h before staining with with anti‐CD3 APC Cy7 (clone OKT3), anti‐CD8 BV650 (clone SK1) and anti-CD4 PB (clone OKT4). Then the cells were fixed with fixation buffer and permeabilized with perm wash buffer (Biolegend, USA) and stained for IFN-γ APC (clone 4S. B3). Live/Dead fixable aqua dead cell stain (Thermo Fisher Scientific, USA) was used according to the manufacturer's protocol to exclude dead cells. Cells were acquired on a BD FACSAria III Cell Sorter using DIVA v8 software (BD Biosciences, USA). For each donor, unstimulated cells were included as a negative control. Flow cytometry data were analyzed using FlowJo v.10.7.1 software (FlowJo). Fluoresces Minus One (FMO) controls were used to draw the gates for both CD107a and IFN-γ (Supplementary Figure 2). The proportion of cells expressing S pool of peptide specific CD107a or producing IFNγ, was determined by subtracting the expression levels/production levels in the unstimulated wells, from the peptide stimulated wells.

### B cell ELISpot assays

Briefly, freshly isolated PBMCs were stimulated in a 24 well plate using IL-2 and R848 (a TLR 7/8 agonist) in RPMI supplemented with 10% fetal bovine serum, 1% penicillin streptomycin and 1% glutamine at 4 million cells/well and incubated at 37 °C with 5% CO_2_ for 3 days. They were then washed and rested overnight and 100,000 cells/well were added. 50,000 cells/well were added to the positive control wells. A Human IgG ELISpot kit (Mabtech 3850-2A) was used according to the manufacturer’s instructions to quantify IgG-secreting cells specific to SARS-COV2 S1, S2 and N recombinant proteins, which were coated at 2 µg/ml in phosphate buffered saline (PBS). All experiments were carried out in duplicate and anti-human IgG monoclonal capture antibodies, was used as a positive control, and media alone as a negative control. The spots were enumerated using an automated ELISpot reader (AID Germany). A positive response was defined as mean ± 2 SD of the background responses. An example of a B cell ELISpot assay for S1, S2 and N recombinant proteins is shown in Supplementary Figure 3.

### Statistical analysis

95% confidence intervals for each category were calculated using the R software (version 4.0.3) and R-studio (version 1.4.1106). Pearson Chi Square Association tests were performed at a confidence level of 95% using the R software in order to identify the statistically significant associations of the age categories and the sex of study participants with antibody responses at 4 weeks. GraphPad Prism version 6 was used for other statistical analysis. In instances when the data were not paired, the Mann–Whitney U test (two tailed) was used and the Wilcoxon matched-pairs signed rank test was used when comparing paired data. The Kruskal–Wallis test was used to compare the differences of the antibody levels in different age groups. Spearman rank order correlation coefficient was used to evaluate the correlation between variables including the association between RBD antibodies, SARS-CoV-2 antibodies, SARS-CoV-2-specific T cell responses and age.

## Supplementary Information

Supplementary Information 1.

Supplementary Information 2.

## References

[1] Medicine, J. H. U. a. *Coronavirus Resource Centre*, https://coronavirus.jhu.edu/ (2021).

[2] Sadarangani M, Marchant A, Kollmann TR (2021). Immunological mechanisms of vaccine-induced protection against COVID-19 in humans. Nat. Rev. Immunol..

[3] Sadoff J (2021). Safety and efficacy of single-dose Ad26.COV2.S vaccine against Covid-19. N. Engl. J. Med..

[4] Ramasamy MN (2021). Safety and immunogenicity of ChAdOx1 nCoV-19 vaccine administered in a prime-boost regimen in young and old adults (COV002): a single-blind, randomised, controlled, phase 2/3 trial. Lancet.

[5] Logunov DY (2021). Safety and efficacy of an rAd26 and rAd5 vector-based heterologous prime-boost COVID-19 vaccine: An interim analysis of a randomised controlled phase 3 trial in Russia. Lancet.

[6] Logunov DY (2020). Safety and immunogenicity of an rAd26 and rAd5 vector-based heterologous prime-boost COVID-19 vaccine in two formulations: two open, non-randomised phase 1/2 studies from Russia. Lancet.

[7] Jones I, Roy P (2021). Sputnik V COVID-19 vaccine candidate appears safe and effective. Lancet.

[8] Voysey M (2021). Safety and efficacy of the ChAdOx1 nCoV-19 vaccine (AZD1222) against SARS-CoV-2: an interim analysis of four randomised controlled trials in Brazil, South Africa, and the UK. Lancet.

[9] Fund, R. D. I. *SPUTNIK V AUTHORIZED IN 30 COUNTRIES*, https://sputnikvaccine.com/newsroom/pressreleases/sputnik-v-authorized-in-30-countries/ (2021).

[10] Fund, R. D. I. *Sputnik Light Covid Vaccine Shows 78.6–83.7% Efficacy Among Elderly: Russian Maker*, https://rdif.ru/Eng_fullNews/6969/ (2021).

[11] Zhang S (2013). Seroprevalence of neutralizing antibodies to human adenoviruses type-5 and type-26 and chimpanzee adenovirus type-68 in healthy Chinese adults. J. Med. Virol..

[12] Barouch DH (2011). International seroepidemiology of adenovirus serotypes 5, 26, 35, and 48 in pediatric and adult populations. Vaccine.

[13] Jeewandara C (2021). Immune responses to a single dose of the AZD1222/Covishield vaccine in health care workers. Nat. Commun..

[14] Jeewandara C (2021). Antibody and T cell responses to a single dose of the AZD1222/Covishield vaccine in previously SARS-CoV-2 infected and naïve health care workers in Sri Lanka. medRxiv.

[15] Tan CW (2020). A SARS-CoV-2 surrogate virus neutralization test based on antibody-mediated blockage of ACE2-spike protein-protein interaction. Nat. Biotechnol..

[16] Jeewandara C (2021). SARS-CoV-2 neutralizing antibodies in patients with varying severity of acute COVID-19 illness. Sci. Rep..

[17] Jeewandara C (2021). Antibody and T cell responses to Sinopharm/BBIBP-CorV in naïve and previously infected individuals in Sri Lanka. medRxiv.

[18] Townsend A (2020). A haemagglutination test for rapid detection of antibodies to SARS-CoV-2. Nat. Commun..

[19] Kamaladasa A (2021). Comparison of two assays to detect IgG antibodies to the receptor binding domain of the SARSCoV2 as a surrogate marker for assessing neutralizing antibodies in COVID-19 patients. Int. J. Infect. Dis..

[20] Pietrobon AJ, Teixeira FME, Sato MN (2020). Immunosenescence and inflammaging: Risk factors of severe COVID-19 in older people. Front. Immunol..

[21] Jeewandara C (2021). Persistence of antibody and T cell responses to the Sinopharm/BBIBP-CorV vaccine in Sri Lankan individuals. medRxiv.

[22] Bosnjak B (2021). Low serum neutralizing anti-SARS-CoV-2 S antibody levels in mildly affected COVID-19 convalescent patients revealed by two different detection methods. Cell. Mol. Immunol..

[23] Khoury DS (2021). Neutralizing antibody levels are highly predictive of immune protection from symptomatic SARS-CoV-2 infection. Nat. Med..

[24] Bergwerk M (2021). Covid-19 Breakthrough Infections in Vaccinated Health Care Workers. N. Engl. J. Med..

[25] Voysey M (2021). Single-dose administration and the influence of the timing of the booster dose on immunogenicity and efficacy of ChAdOx1 nCoV-19 (AZD1222) vaccine: a pooled analysis of four randomised trials. Lancet.

[26] Lopez Bernal J (2021). Effectiveness of Covid-19 vaccines against the B.1.617.2 (Delta) variant. N. Engl. J. Med..

[27] Coughlan L (2020). Factors which contribute to the immunogenicity of non-replicating adenoviral vectored vaccines. Front. Immunol..

[28] Buchbinder SP (2008). Efficacy assessment of a cell-mediated immunity HIV-1 vaccine (the Step Study): A double-blind, randomised, placebo-controlled, test-of-concept trial. Lancet.

[29] Frahm N (2012). Human adenovirus-specific T cells modulate HIV-specific T cell responses to an Ad5-vectored HIV-1 vaccine. J. Clin. Investig..

[30] Madhi SA (2021). Efficacy of the ChAdOx1 nCoV-19 Covid-19 Vaccine against the B.1.351 Variant. N. Engl. J. Med..

[31] Sette A, Crotty S (2021). Adaptive immunity to SARS-CoV-2 and COVID-19. Cell.

[32] Ewer KJ (2021). T cell and antibody responses induced by a single dose of ChAdOx1 nCoV-19 (AZD1222) vaccine in a phase 1/2 clinical trial. Nat. Med..

[33] Galipeau Y, Greig M, Liu G, Driedger M, Langlois MA (2020). Humoral responses and serological assays in SARS-CoV-2 infections. Front. Immunol..

[34] Zhou D (2020). Structural basis for the neutralization of SARS-CoV-2 by an antibody from a convalescent patient. Nat. Struct. Mol. Biol..

[35] Malavige GN (2008). Viral load, clinical disease severity and cellular immune responses in primary varicella zoster virus infection in Sri Lanka. PLoS ONE.

[36] Peng Y (2020). Broad and strong memory CD4(+) and CD8(+) T cells induced by SARS-CoV-2 in UK convalescent individuals following COVID-19. Nat. Immunol..

