# Supplementary material for: Immune responses following the first dose of the Sputnik V (Gam-COVID-Vac)
Source: Sci Rep. 2022 Feb 2;12:1727. doi: 10.1038/s41598-022-05788-6 (PMC8810924; doi:10.1038/s41598-022-05788-6)
Supplement: Supplementary file 1 — Supplementary Information 1. [file 41598_2022_5788_MOESM1_ESM.docx]

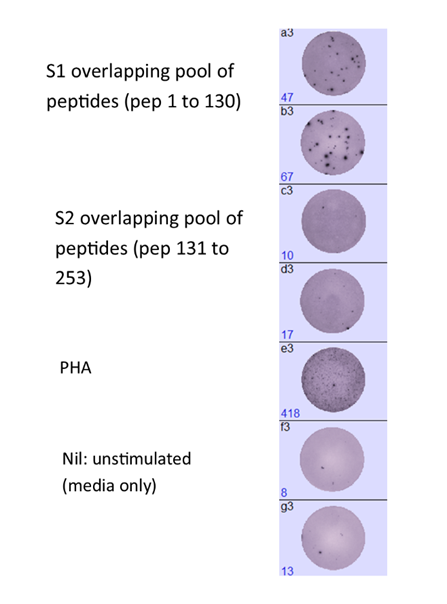

 A B


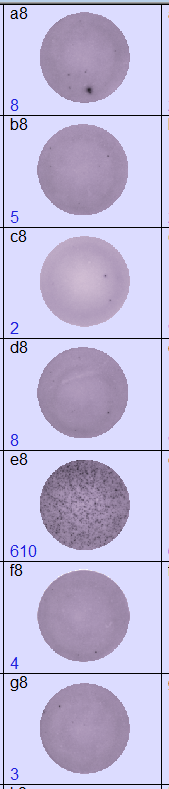


**Supplementary figure 1**: An example of an ex vivo ELISpot response

1. at 4 weeks following the Gam-COVID-Vac
2. Baseline uninfected


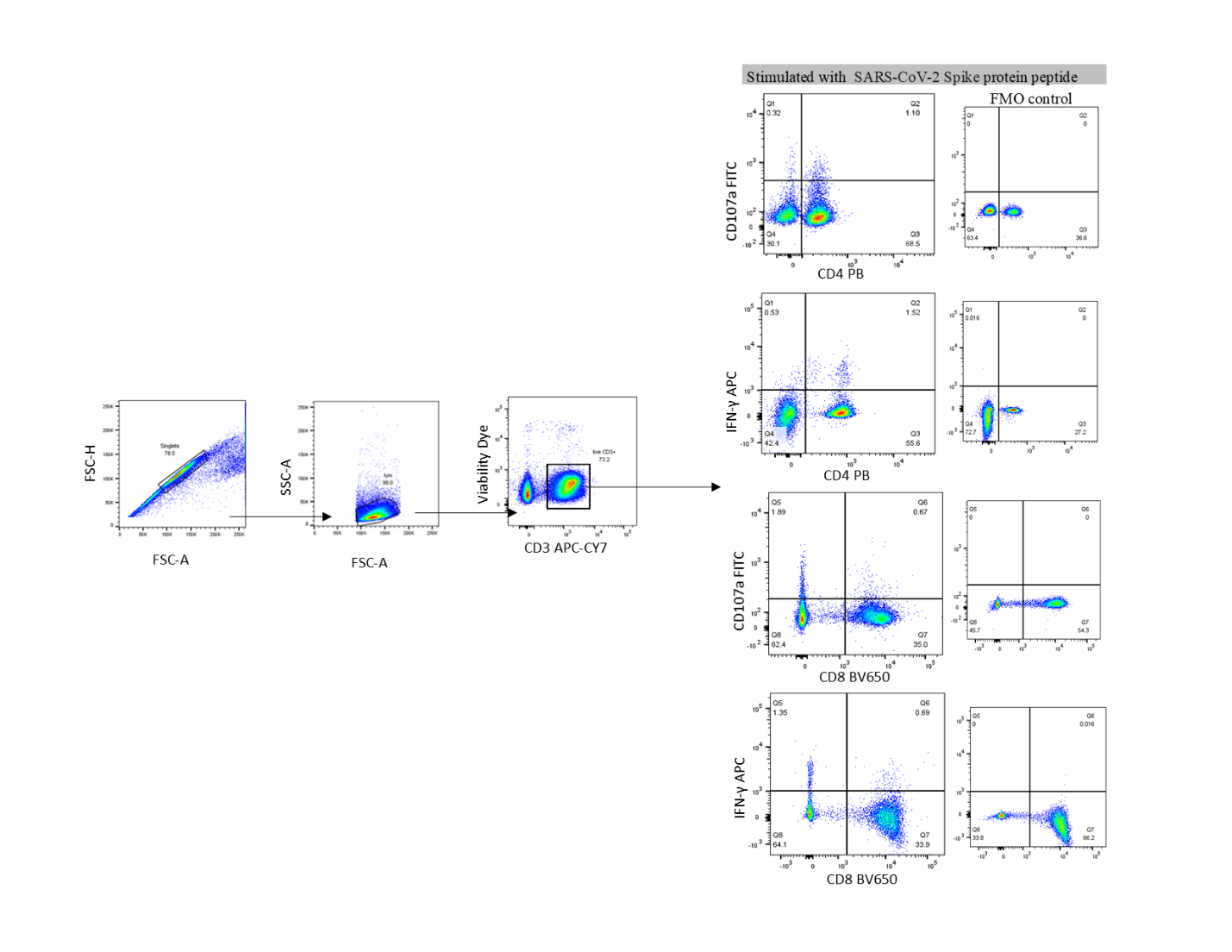


**Supplementary figure 2:** Gating strategy used to identify CD107a expressing CD4+ and CD8+ T cells and IFNγ producing CD4+ and CD8+ T cells. The cells were initially gates on FSC-H and FSC-A to gate the singlets. The lymphocytes from these cells were then identified by gating them on the FSC and SSC. From these cells, the live cells were then gates and then CD3+ T cells were gated. From these CD3+ T cells, CD107a expressing CD4+ and CD8+ T cells were identified and IFNγ producing CD4+ T cells and CD8+ T cells were identified.


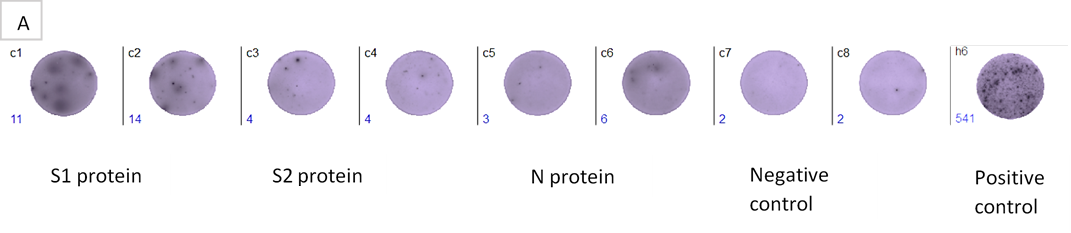

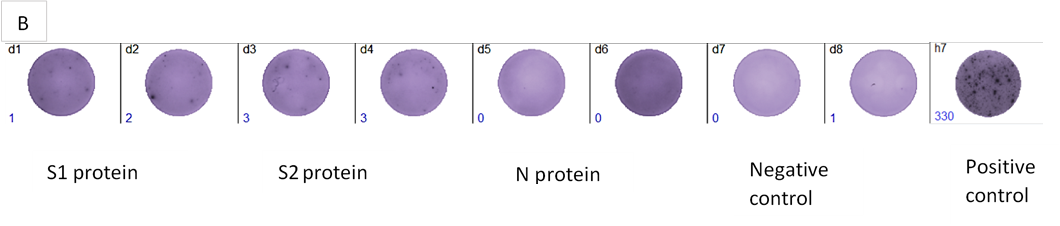


**Supplementary figure 3:** An example of a B cell ELISpot assay response

1. At 4 weeks following the Gam-COVID-Vac for S1, S2 and N recombinant protein.
2. Baseline uninfected
