# Supplementary material for: Immune responses following the first dose of the Sputnik V (Gam-COVID-Vac)
Source: Sci Rep. 2022 Feb 2;12:1727. doi: 10.1038/s41598-022-05788-6 (PMC8810924; doi:10.1038/s41598-022-05788-6)
Supplement: Supplementary file 2 — Supplementary Information 2. [file 41598_2022_5788_MOESM2_ESM.docx]

|  | **Total n = 327** | | **Positive (290, 88.69%)** | | **Negative (37, 11.31%)** | |
| --- | --- | --- | --- | --- | --- | --- |
|  | **Mean /**  **Frequency** | **SEM /**  **Percentage** | **Mean /**  **Frequency** | **SEM /**  **Percentage** | **Mean /**  **Frequency** | **SEM /**  **Percentage** |
| **Gender** |  |  |  |  |  |  |
| Females | 203 | 62.08% | 185 | 91.13% | 18 | 8.87% |
| Males | 124 | 37.92% | 105 | 84.68% | 19 | 15.32% |
| **Age** | 50.29 | 0.78 | 49.33 | 0.83 | 57.84 | 2.02 |
| 20-39 | 71 | 21.71% | 70 | 98.59% | 01 | 1.41% |
| 40-59 | 168 | 51.38% | 148 | 88.10% | 20 | 11.90% |
| ≥ 60 | 88 | 26.91% | 72 | 81.82% | 16 | 18.18% |
| **Comorbidities** |  |  |  |  |  |  |
| diabetes | 52 | 15.90% | 43 | 14.83% | 09 | 24.32% |
| hypertension | 47 | 14.37% | 40 | 13.79% | 07 | 18.92% |
| chronic kidney disease | 01 | 0.31% | 01 | 0.34% | 00 | 0.00% |
| asthma | 16 | 4.89% | 15 | 5.17% | 01 | 2.70% |
| Individuals with more than one comorbidity | 26 | 7.95% | 22 | 7.59% | 04 | 10.81% |

**Supplementary table 1: Demographic details and comorbidities of study participants**
